# Supplementary figures and images for: Gene regulatory network architecture in different developmental contexts influences the genetic basis of morphological evolution
Source: PLoS Genet. 2018 May 3;14(5):e1007375. doi: 10.1371/journal.pgen.1007375 (PMC5953500; doi:10.1371/journal.pgen.1007375)

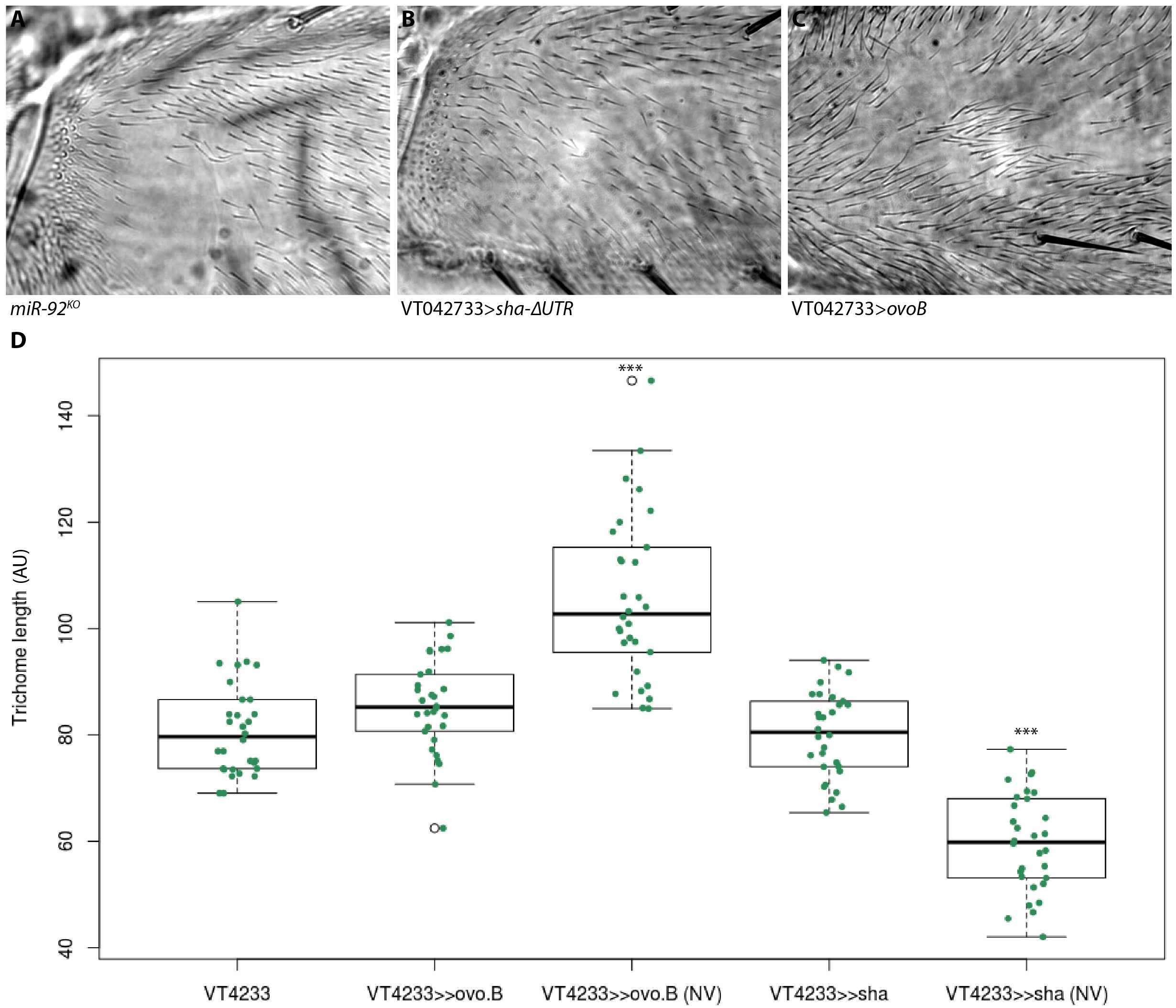

Supplement: S1 Fig — (A) Trichomes gained in the naked valley after loss of miR-92a and miR-92b have a similar morphology as trichomes on the more distal femur. Trichomes gained after ectopic expression of sha-ΔUTR (B) are significantly shorter, while trichomes developing after expression of ovoB (C) are significantly longer than on the remaining femur. (D) Trichomes on the more distal femur have a similar length as in the driver line (VT42733) regardless of whether ovoB or sha are expressed under its control, but trichomes gained in the naked valley are significantly longer or shorter, respectively (p<0.001). Tukey’s multiple comparison test was used to test for significance. (JPG) [file pgen.1007375.s001.jpg]

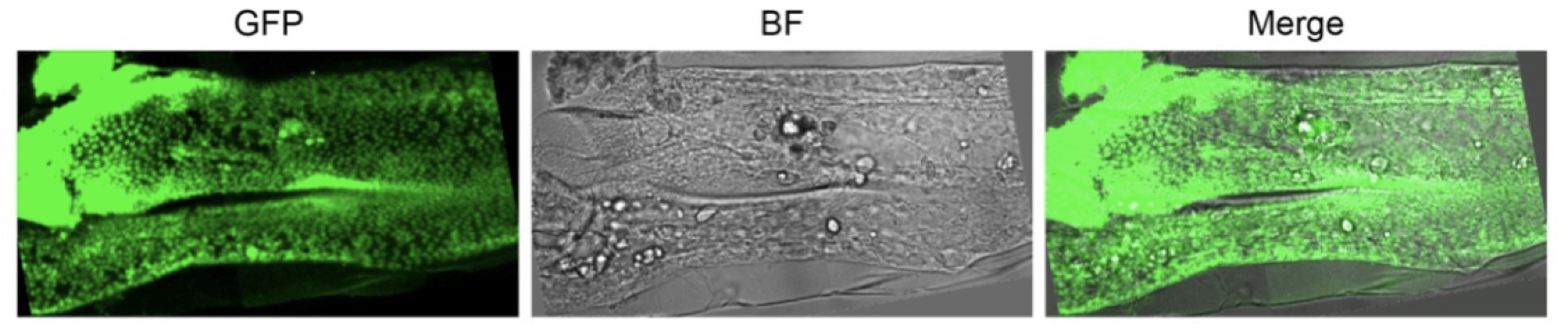

Supplement: S2 Fig — GFP is expressed throughout the posterior femur of a T2 leg at 24 hAPF. (JPG) [file pgen.1007375.s002.jpg]

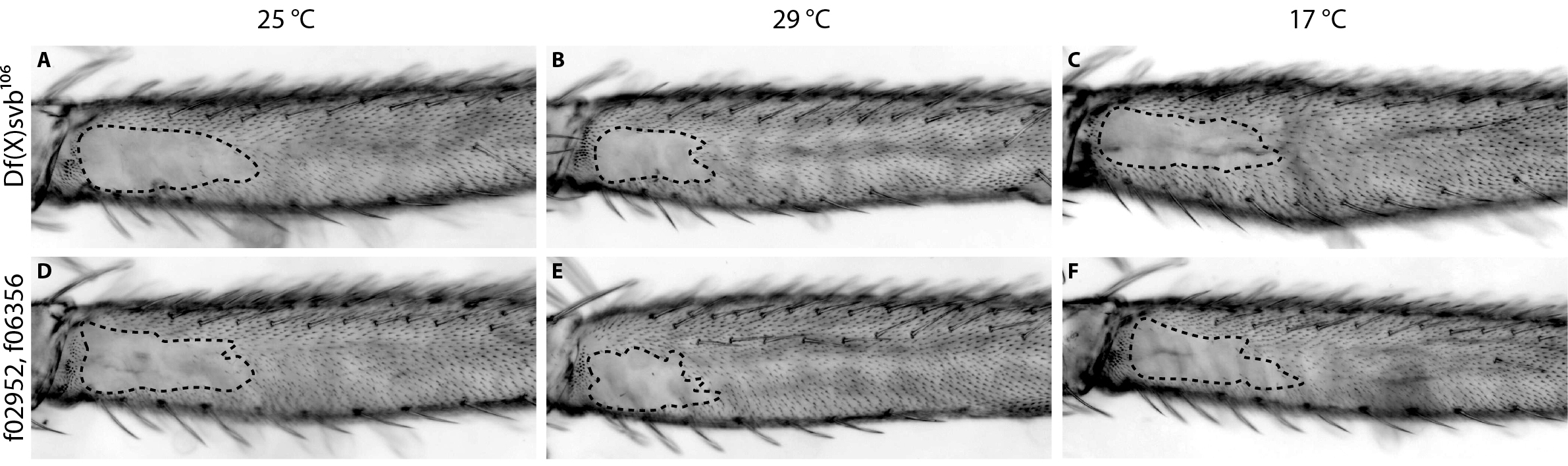

Supplement: S3 Fig — The control line still contains both pBac insertions used to generate the deficiency [5,43]. There is no detectable difference in naked valley size or trichome density between deficiency and control flies at 25°C, 29°C, or 17°C. (JPG) [file pgen.1007375.s003.jpg]

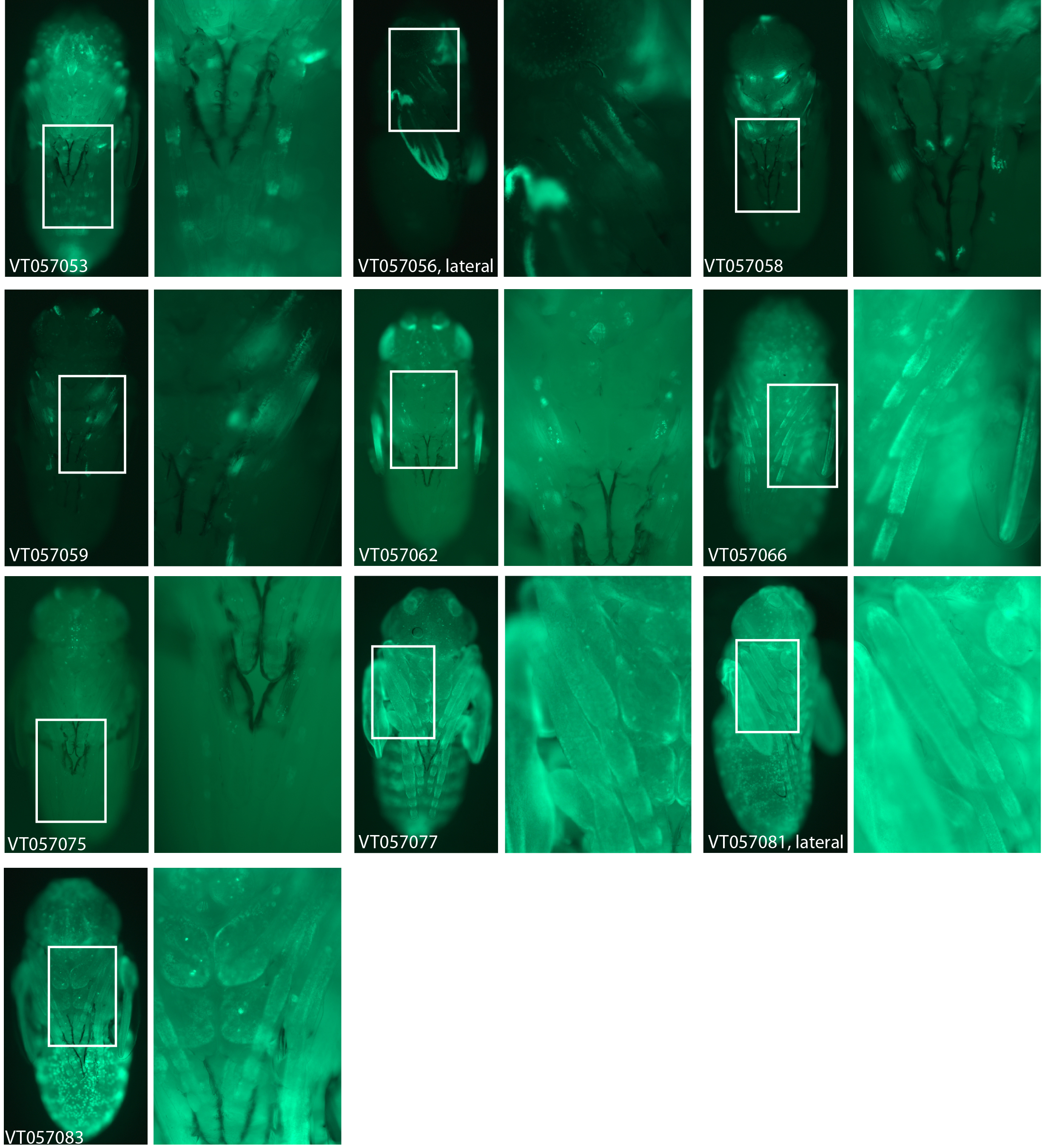

Supplement: S4 Fig — All tested drivers show some expression in T2 legs as well as in other pupal tissues. (JPG) [file pgen.1007375.s004.jpg]

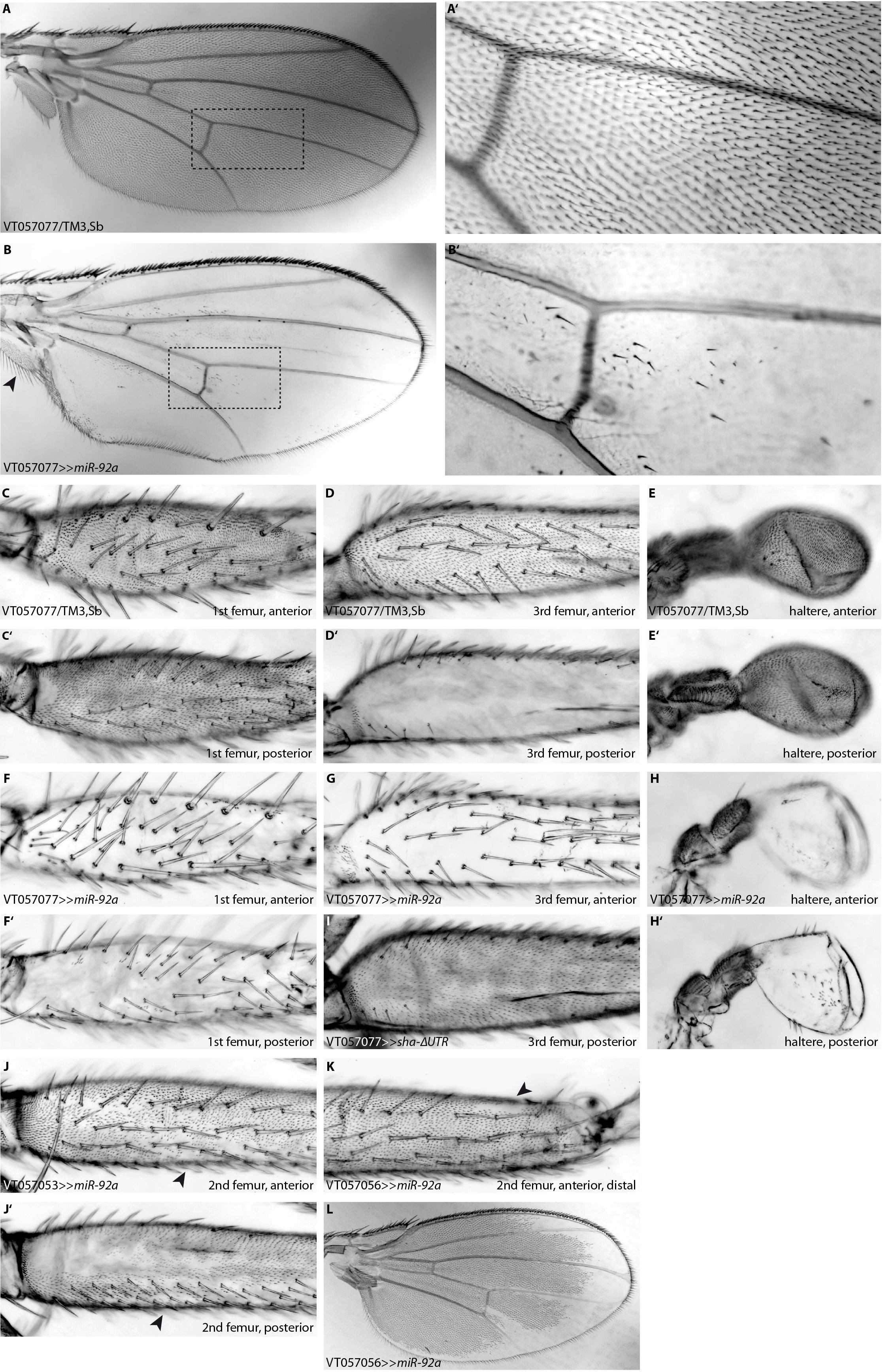

Supplement: S5 Fig — (A, A’, B, B’) Trichomes on the wing are largely repressed upon expression of miR-92a under control of VT057077. Note that trichomes on the alula (arrowhead in B) develop normally. Also trichomes on T1 and T3 legs (C, C’ D, F, F’, G) and on the halteres (E, E’, H, H’) are repressed when miR-92a is driven by VT057077. (I) Driving sha-ΔUTR under control of VT057077 leads to ectopic formation of trichomes on the posterior T3 leg (compare to D’). (J, J’) Trichomes on the ventral side of the femur are partially repressed when miR-92a is expressed under control of VT057053. Trichomes are repressed in a patch on the dorsal side of the distal T2 femur (K) and around the rim of the distal wing (L) after expression of miR-92a under control of VT057056. (JPG) [file pgen.1007375.s005.jpg]

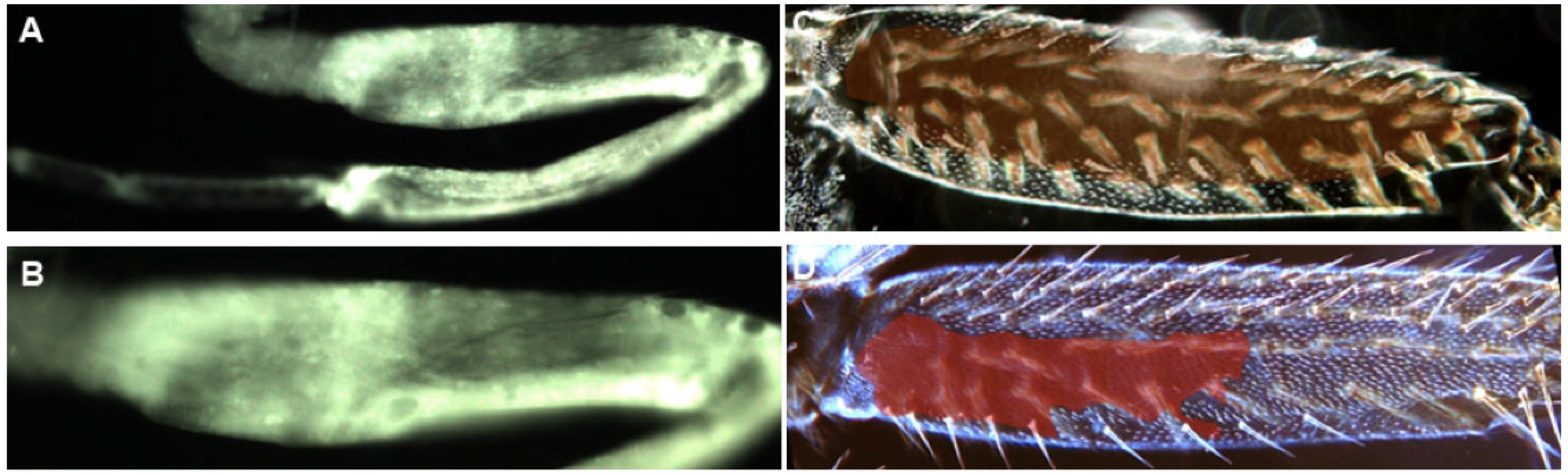

Supplement: S6 Fig — GFP is expressed throughout all the leg segments (A) including the femur (B) of the second leg. Mutant clones of tals18 (C) (brown shaded area) and svbR9 (D) (red shaded area) lack trichomes on the femur of a second leg. (JPG) [file pgen.1007375.s006.jpg]

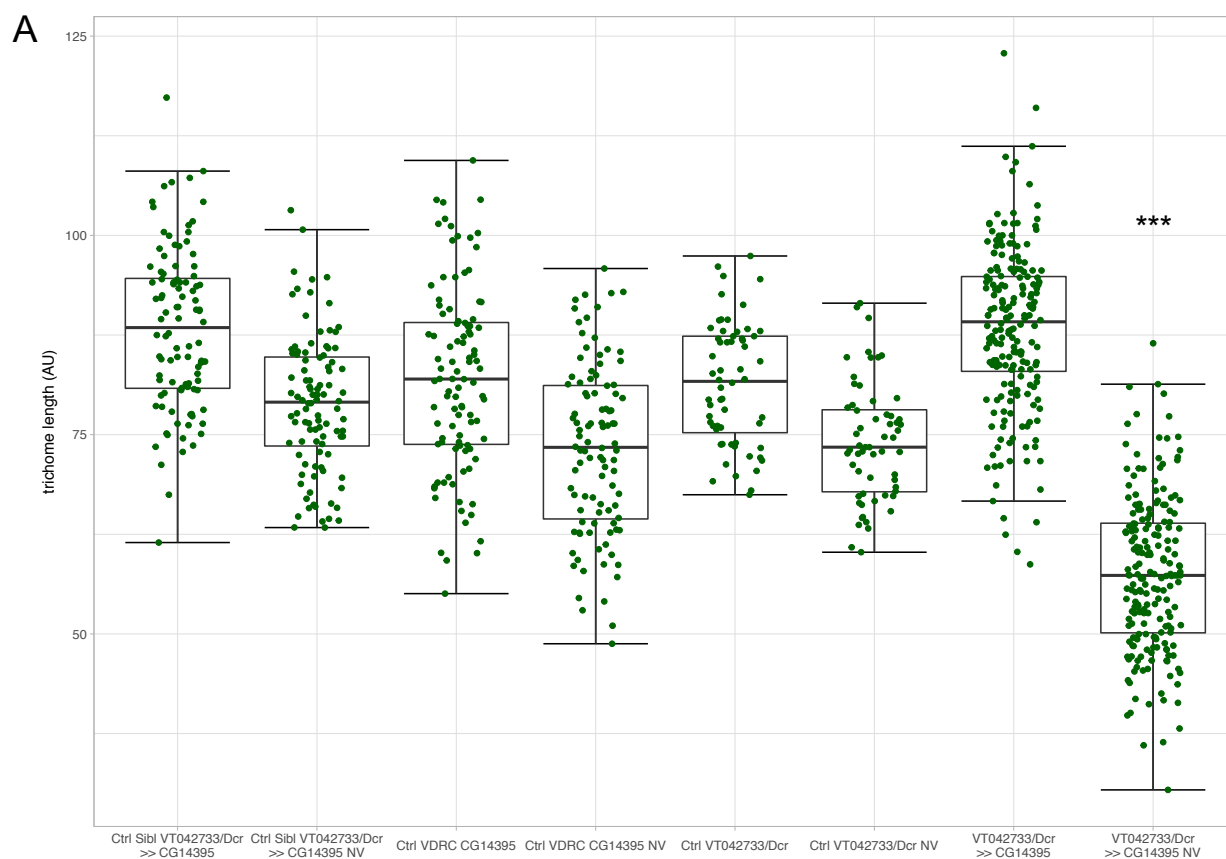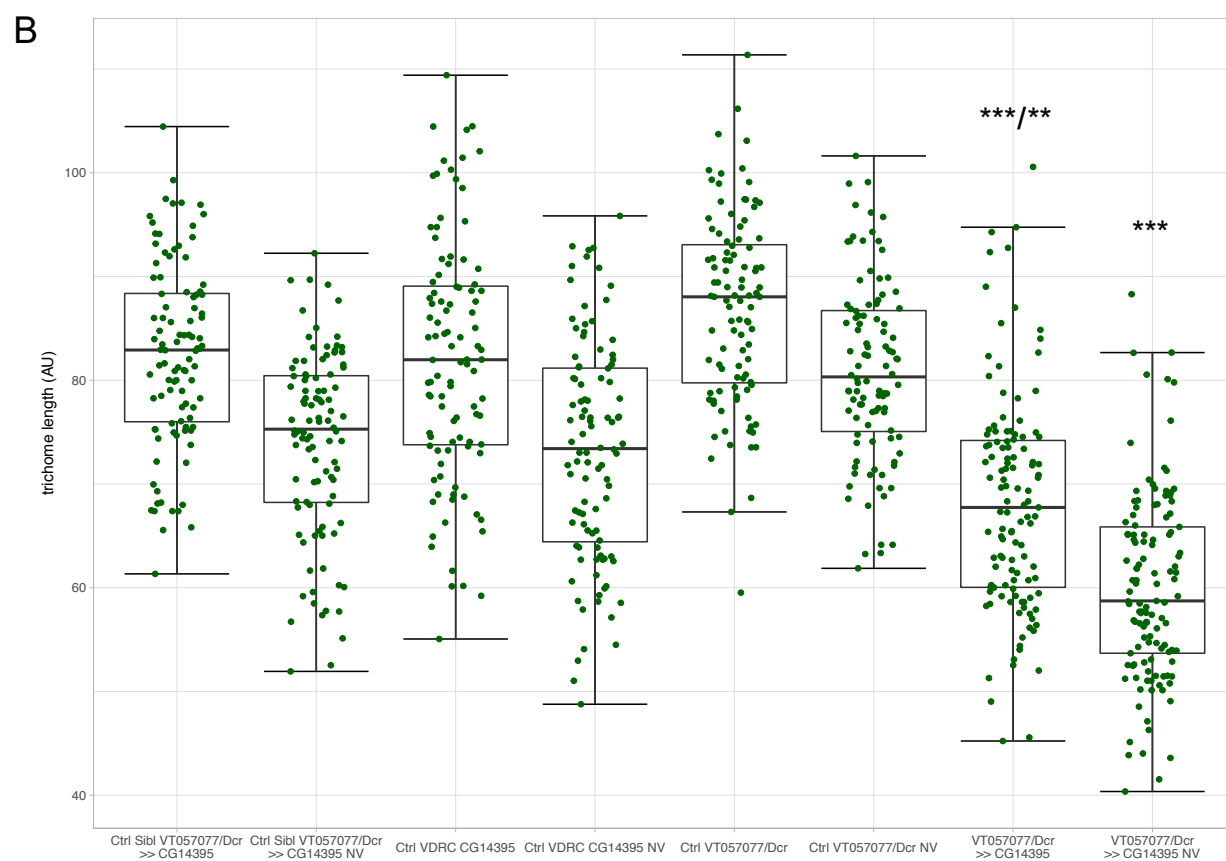

Supplement: S7 Fig — Expression of the RNAi construct and UAS-Dicer was under control of GAL4 driver lines VT042733 (drives in the proximal femur) and VT057077 (drives in the whole leg). Box plots show the length of trichomes in the distal part of the posterior femur and around the naked valley (NV). Parents (UAS-Dcr/CyO;VT042733/TM6B or UAS-Dcr/CyO;VT057077/TM6B females, VDRC CG14395-RNAi males) and siblings without knockdown effect were used as controls (Ctrl). (A) Trichomes developing after knockdown of CG14395 in the proximal femur are significantly shorter around the naked valley area than on the remaining femur (distal part) and on femurs of the controls (p < 0.001). Data are normally distributed (Shapiro-Wilk normality test). Tukey’s multiple comparison test was used to test for significance. (B) After knockdown of CG14395 in the whole leg, trichomes are significantly shorter both around the naked valley area and on the remaining femur (p < 0.001 and p < 0.01). Note that some controls show significantly different trichome lengths. Data are not normally distributed (Shapiro-Wilk normality test). Kruskal-Wallis and pairwise comparisons using Wilcoxon rank sum test were used to test for significance. (PDF) [file pgen.1007375.s007.pdf]
